# Designed Antimicrobial Peptides for Recurrent Vulvovaginal Candidiasis Treatment

**DOI:** 10.1128/AAC.02690-18

**Published:** 2019-10-22

**Authors:** Kathryn W. Woodburn, L. Edward Clemens, Jesse Jaynes, Lydia-Marie Joubert, Alfred Botha, Hasan Nazik, David A. Stevens

**Affiliations:** a Riptide Bioscience, Inc., Vallejo, California, USA; b Integrative Biosciences, College of Agriculture, Environment and Nutrition Sciences, Tuskegee University, Tuskegee, Alabama, USA; c Microbiology Department, Stellenbosch University, Stellenbosch, South Africa; d Central Analytical Facilities, Stellenbosch University, Stellenbosch, South Africa; e California Institute for Medical Research, San Jose, California, USA; f Division of Infectious Diseases and Geographic Medicine, Stanford University, Stanford, California, USA

**Keywords:** antimicrobial peptides, antifungals, vulvovaginal candidiasis, biofilm, antifungal activity, fungicidal activity

## Abstract

Recurrent vulvovaginal candidiasis (RVVC) is a widespread chronic infection that has a substantial negative impact on work and quality of life. The development of antimicrobial resistance and biofilm formation are speculated to contribute to *Candida* pathogenicity and treatment ineffectiveness. Designed antimicrobial peptides (dAMPs) are chemically modified from endogenous antimicrobial peptides that provide the first line of defense against pathogens. The goal here is to identify a dAMP for the topical treatment of RVVC. The dAMP MICs were determined for 46 fluconazole-susceptible and fluconazole-resistant *Candida* spp. clinical isolates. The possibility of inducing dAMP drug resistance and comparison of dAMP and fluconazole activity against preformed *Candida* biofilm and biofilm formation were evaluated. Assessment of mammalian cell viability was determined using bioluminescent human keratinocytes. The dAMP effect on fungus was probed via scanning electron microscopy, and topically applied dAMP activity was evaluated in a rodent vulvovaginal candidiasis (VVC) infection model. dAMPs demonstrated broad-spectrum antimicrobial activity against common causative clinical *Candida* isolates, reduced preformed biofilm, and inhibited biofilm formation. An evaluated dAMP did not induce resistance after repeated exposure of Candida tropicalis. The dAMPs were selective for *Candida* cells with limited mammalian cytotoxicity with substantial activity in a rodent VVC model. dAMPs are described as having potent antifungal and antibiofilm activity, likely direct membrane action with selectivity for *Candida* cells, with limited resistance development. Combined with activity in a rodent VVC model, the data support clinical evaluation of dAMPs for topical treatment of VCC and recurrent VVC infections.

## Abstract (precis)

Recurrent vulvovaginal candidiasis (RVVC) is a widespread chronic infection that has a substantial negative impact on work and quality of life. The development of antimicrobial resistance and biofilm formation are speculated to contribute to *Candida* pathogenicity and treatment ineffectiveness. Designed antimicrobial peptides (dAMPs) are chemically modified from endogenous antimicrobial peptides that provide the first line of defense against pathogens.

## INTRODUCTION

Recurrent vulvovaginal candidiasis (RVVC), defined as four or more vulvovaginal candidiasis (VVC) infections per year, is a widespread chronic, often drug-resistant mucosal infection that has a substantial negative impact on work and quality of life (1–3) and annually affects approximately 138 million women worldwide (4, 5). RVVC infection is caused by Candida albicans and non-*albicans Candida* species (4). VVC and RVVC are often treated with azole agents that are fungistatic due to inhibition of ergosterol biosynthesis in *Candida* spp. (6); however, they are substantially less active against non-*albicans Candida* spp. (2, 7).

For RVVC treatment, the Infectious Diseases Society of America guidelines recommend a long-term off-label suppressive treatment regimen with oral fluconazole for at least 6 months (8); however, recurrence rates of 60% to 70% are observed (9, 10). There are no products currently approved for the treatment of RVVC. Additionally, most infections occur in women of childbearing potential. The FDA, due to teratogenicity concerns (11), has advised against prescribing oral fluconazole for VCC treatment during pregnancy.

The development and spread of antimicrobial resistance and the shift in yeast epidemiology, from C. albicans to non-*albicans Candida* spp., including Candida glabrata, Candida dubliniensis, Candida lusitaniae, Candida tropicalis, Candida krusei, Candida kefyr, and Candida parapsilosis, have been driven by chronic use of over-the-counter azoles (4, 12). Furthermore, to exacerbate RVVC recalcitrance, *Candida* could form drug-resistant biofilm (4, 13, 14). Fungal biofilms are complex colonies of microorganisms encased in a dense extracellular matrix comprised of proteins, polysaccharides, and carbohydrates, thereby providing protection from the host’s immune system and antifungal agents (15, 16). For RVVC, innovation in antifungal treatment is needed due to the rapid emergence of fungal resistance, a recent shift in increased epidemiologic prevalence of fluconazole-resistant non-*albicans* species, and the possible difficulty of treating biofilms.

Designed antimicrobial peptides (dAMPs) are laboratory-synthesized peptides that have been rationally designed from naturally occurring antimicrobial peptides (AMPs) that provide defense against invading pathogens (17). AMPs have direct antipathogenic activities and kill fungi as well as both Gram-negative and Gram-positive bacteria (18–20). An important action of AMPs is direct membranolytic electrostatic perturbation of the barrier function of the pathogen’s membrane (21), with cell selectivity increased by the presence of ergosterol, a membrane lipid found in fungi (22). Moreover, this remarkable targeting and direct contact disruption of the pathogen’s membrane makes resistance less likely to develop (18), an integral feature for fighting antimycotic-resistant infections. Four dAMPs rationally derived from tachyplesin I, a disulfide β-sheet antimicrobial peptide, from the horseshoe crab (Tachypleus tridentatus), were evaluated for their potential utility to topically treat RVVC.

## RESULTS

### *In vitro* antifungal activity.

The dAMPs (Table 1) exhibited broad-spectrum antimicrobial activity against *Candida* strains (Table 2), including against the intrinsically fluconazole-resistant C. krusei. The MICs of fluconazole-susceptible and resistant C. albicans and C. glabrata and resistant C. krusei and C. parapsilosis were 16 to >32 μg/ml corresponding to 6.5 to >17 μM, though a minority of the total tested for RP556 resulted in an MIC of 8 μg/ml (3.7 μM). In contrast, the dAMP MICs of fluconazole-susceptible and resistant C. tropicalis, susceptible C. parapsilosis, C. kefyr, C. lusitaniae, C. dubliniensis, Candida sphaerica, and Candida famata were largely 2 to 8 μg/ml (0.81 to 4.3 μM). In the fluconazole-resistant strains, the dAMPs were more effective than fluconazole, based on μM levels, in all of the *Candida* spp. tested. In terms of a microgram per milliliter comparison, fluconazole was more effective in only C. albicans and C. parapsilosis.

**TABLE 1:** dAMPs evaluated in support of RVVC treatment evaluation

Peptide	Amino acid sequence	Length (aa)^*a*^
RP504	FOIOAOLGGCLGOFCGGIOAOLOF-NH_2_; disulfide bridge: C_10_–C_15_	24
RP554	FOLOAOIOVOLOAOIOL-NH_2_	17
RP556	RWCFKVCYKGICYKKCK-NH_2_; disulfide bridge: C_3_–C_16_, C_7_–C_12_	17
RP557	RFCWKVCYKGICFKKCK-NH_2_; disulfide bridge: C_3_–C_16_, C_7_–C_12_	17

a aa, amino acids.

**TABLE 2:** MICs against fluconazole-resistant and sensitive *Candida*

*Candida* species	MIC for (μM [μg/ml]):^*c*^				
	RP504	RP554	RP556	RP557	Fluconazole^*a*^
Resistant					
C. albicans	6.5 (1), 13 (2), >13 (7) [16 (1), 32 (2), >32 (7)]	8.5 (4), 17 (5), >17 (1) [16 (4), 32 (5), >32 (1)]	3.7 (2), 7.4 (5), 14.8 (3) [8 (2), 16 (5), 32 (3)]	7.5 (2), 15 (5), >15 (3) [16 (2), 32 (5), >32 (3)]	≥26 [≥8]
C. glabrata	>13 (4) [>32 (4)]	17 (3), >17 (1) [32 (3), >32 (1)]	15 (4) [32 (4)]	15 (2), >15 (2) [32 (2), >32 (2)]	≥209 [≥64]
C. tropicalis	1.6 (2) [4 (2)]	1.1 (1), 2.1 (1) [2 (1), 4 (1)]	0.93 (2) [2 (2)]	1.9 (2) [4 (2)]	≥26 [≥8]
C. parapsilosis	>13 [>32]	17 [32]	15 [32]	>15 [>32]	≥26 [≥8]
C. krusei^*b*^	>13 (3) [>32 (3)]	8.5 (2), 17 (1) [16 (2), 32 (1)]	3.7 (1), 7.4 (2) [8 (1), 16 (2)]	7.5 (1), 15 (2) [16 (1), 32 (2)]	
Susceptible					
C. albicans	13 (5) [32 (5)]	8.5 (4), 17 (1) [16 (4), 32 (1)]	7.4 (4), 15 (1) [16 (4), 32 (1)]	15 (4), >15 (1) [32 (4), >32 (1)]	≤6.5 [≤2]
C. glabrata	13 (1), >13 (4) [32 (1), >32 (4)]	17 (5) [32 (5)]	7.4 (2), 15 (3) [16 (2), 32 (3)]	15 (4), >15 (1) [32 (4), >32 (1)]	
C. tropicalis	1.6 (4), 3.3 (1) [4(4), 8 (1)]	1.1 (1), 2.1 (4) [2 (1), 4 (4)]	0.93 (4), 1.9 (1) [2 (4), 4 (1)]	1.9 (3), 3.7 (2) [4 (3), 8 (2)]	≤6.5 [≤2]
C. parapsilosis	3.3 (1), 13 (1), >13 (3) [8 (1), 32 (1), >32 (3)]	1.1 (1), 2.1 (1), 8.5 (3) [2 (1), 4 (1), 16 (3)]	3.7 (2), 15 (3) [8 (2), 32 (3)]	3.7 (1), 15 (1), >15 (3) [8 (1), 32 (1), >32 (3)]	≤6.5 [≤2]
C. kefyr	1.6 (1), 3.3 (1) [4 (1), 8 (1)]	2.1 (2) [4 (2)]	0.93 (1), 1.9 (1) [2 (1), 4 (1)]	1.9 (1), 3.7 (1) [4 (1), 8 (1)]	>3.3 [>1]
C. lusitaniae	1.6 [4]	1.1 [2]	3.7 [8]	3.7 [8]	>6.5 [>2]
C. dubliniensis	9.6 [32]	4.3 [8]	3.7 [8]	7.5 [16]	>1.6 [>0.5]
*C. sphaerica*	6.5 [16]	4.3 [8]	1.9 [4]	3.7 [8]	
C. famata	3.3 [8]	2.1 [4]	7.4 [16]	7.5 [16]	

a Clinical breakpoints taken from 2017 CLSI recommendations (37) and Pfaller and Diekema (42). Molecular weights are as follows: RP504, 2,455 amu; RP554, 1,884 amu; RP556, 2,157 amu; RP557, 2,136 amu; and fluconazole, 306 amu.

b Isolates of C. krusei are intrinsically resistant to fluconazole (37).

c Inhibition of planktonic growth was assessed using current CLSI methodology (37). Numbers in parenthesis indicate the number of strains with the MICs shown.

Minimum fungicidal concentrations (MFCs) were determined on 9 isolates, representatives of selected groups as listed in Materials and Methods, each tested against all 4 dAMPs (total, 36 assays). In 14 of the 36 assays, the MFC was the same as the MIC or slightly higher. In 3 assays, the MFC was 2 tube dilutions higher than the MIC. In 4 assays, the MICs ranged from 4 to 16 μg/ml, with MFCs of >32 μg/ml. In 7 assays, the MIC was 32 μg/ml, and the MFC was >32 μg/ml. In 8 assays, the MFC/MIC ratio could not be determined, as the MIC was >32 μg/ml. Thus, most commonly, the MIC and MFC values of the dAMPs were near to each other in value, suggesting that the inhibiting mechanism is commonly a lethal event, and showed fungicidal activity for the dAMPs.

Classically, fluconazole is regarded as only a fungistatic agent, and this was confirmed with C. albicans (17-88), where the MIC was 1 μg/ml (3.27 μM) and the MFC was >64 μg/ml (>209 μM). This contrast, with a lack of fluconazole fungicidal activity, was striking. Because of the frequent similarity of dAMP MIC and MFC with representatives of the selected groups (detailed in Materials and Methods), it was of interest then to similarly study fluconazole further. Clinical isolates sent to the California Institute for Medical Research (CIMR) reference laboratory were assayed. Eleven of 11 C. albicans isolates had MFCs >4-fold and up to >64 fold greater than MICs.

### *Candida* did not develop resistance, under the specific experimental conditions used, against RP554, RP556, and RP557.

The starting MICs and MFCs, before the serial passages in increasing concentrations, for RP556, RP557, RP554 were 2, 4, and 4 μg/ml and 2, 4, and 8 μg/ml, respectively. When the passage level of 16 μg/ml was reached, none of the dAMP-passaged C. tropicalis isolates survived for further study. Thus, survivors at the lower level of 8 μg/ml were tested, and the MICs and MFCs for RP556, RP557, RP554 were 2, 4, and 4 μg/ml (0.93, 1.9, 2.1 μM) and 8, 8, and 8 μg/ml (3.7, 3.7, 4.3 μM), respectively. Thus, the MICs for all 3 dAMPs were unchanged by serial passages at increasing concentrations, and the MFCs were unchanged or slightly changed. Thus, for none of the dAMPs did serial passages (total, 9 passages in doubly increasing concentrations) increase the MIC. The concurrent control, passaged 9 times in the absence of drug, had no changes from the starting MIC and MFC.

### Fungal biofilm inhibition.

**FIG 1:**
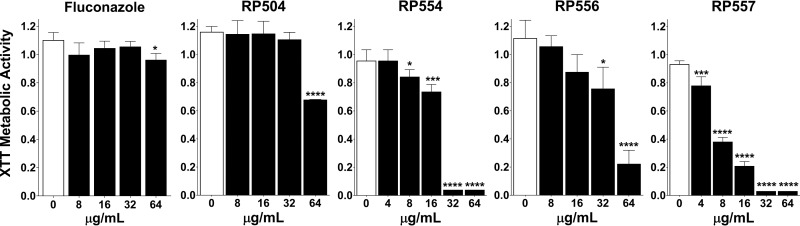
dAMPs effectively inhibit fluconazole-resistant C. albicans preformed fungal biofilm. Fluconazole and dAMPs were added to C. albicans 17-88 preformed biofilm for 24 h, and biofilm inhibition was evaluated via metabolic evaluation using XTT (39). Data represent the mean ± standard deviation (SD) of 3 to 4 measurements, with RP554 and RP557 evaluated in two independent experiments; statistical significance, compared to control, was determined by one-way ANOVA followed by Dunnett’s test (*, *P* < 0.05; ***, *P* < 0.001; ****, *P* < 0.0001).

Biofilm formation is a key driver of C. albicans pathogenicity. The dAMPs were remarkably effective in inhibiting biofilm formation (Table 3) and preformed C. albicans biofilm (example in Fig. 1 and Table 3). RP557 was quite potent with 16 μg/ml (7.5 μM) and 4 μg/ml (1.9 μM) required to inhibit biofilm formation and mature biofilm, respectively. Fluconazole was only marginally inhibitory, even at 64 μg/ml (209 μM). The order of effectiveness (lowest concentration with a statistically significant result) on preformed C. albicans biofilm was as follows: RP557 ≥ RP554 > RP556 ∼> RP504 ⋙ fluconazole; that for inhibiting biofilm growth was as follows: RP557 > RP554 > RP556 ∼ RP504 ⋙ fluconazole (Fig. 1).

**TABLE 3:** Susceptibility of dAMPs and fluconazole against planktonic yeasts, biofilm formation, and preformed biofilm for C. albicans isolate 17-88

Agent	MIC (μM [μg/ml])	Biofilm formation Inhibition^*a*^ (μM [μg/ml])	Biofilm formation activity quotient^*b*^	Preformed biofilm inhibition^*a*^ (μM [μg/ml])	Preformed biofilm activity quotient^*b*^
Fluconazole	3.3 [1.0]	209 [64]	64	209 [64]	64
RP504	13 [32]	>26 [>64]	>2	26 [64]	2
RP554	8.5 [16]	17 [32]	2	4.3 [8]	0.5
RP556	7.4 [16]	15 [32]	2	15 [32]	2
RP557	15 [32]	7.5 [16]	0.5	1.9 [4]	0.125

a Lowest concentration with statistically significant inhibition.

b Ratio of biofilm activity endpoint, statistically significant difference, to MIC.

The dAMPs inhibited established biofilm. Particularly potent were RP557 and RP554 at concentrations substantially less than those required to inhibit planktonic fungi (Table 3). Fluconazole was inhibitory at 64 μg/ml (209 μM) whereas RP557 was quite potent at 4 μg/ml (1.9 μM). The preformed biofilm activity quotient (Table 3) was 0.125 and 0.5 for RP557 and RP554, respectively, in comparison to that for fluconazole, which produced a ratio of 64. The dAMPs were also potent against inhibiting biofilm formation, with activity quotients of 0.5 and 2 for RP557 and RP554, respectively, in comparison to that for fluconazole with a ratio of 64.

### Direct cell wall perturbation.

**FIG 2:**
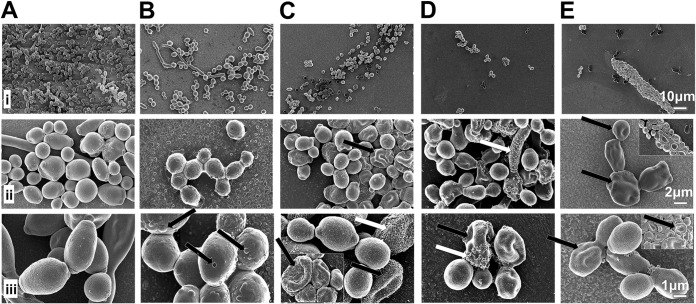
Damage of C. albicans biofilm by RP554. Scanning electron micrographs of C. albicans 17-88 biofilm incubated with test article for 24 h. (A) Control (no treatment); (B) 64 μg/ml fluconazole; (C to E) 16, 32, and 64 μg/ml RP554. Rows i to iii represent micrographs captured at ×1,000, ×5,000, and ×10,000 magnification; refer to scale bars for actual size range. The black arrows indicate cell wall indentations and cell membrane damage, and white arrows indicate surface coating with extracellular matrix residue.

Scanning electron microscopy (SEM) on planktonic and on preformed biofilm C. albicans suggested that dAMP-mediated fungal damage was via membrane perturbation. Control planktonic C. albicans appeared as well-rounded and intact yeasts with prominent bud scars; whereas fluconazole-treated cells had scattered irregular areas across the cell surface, reflecting some compromised cell membranes and walls. The membrane surfaces of dAMP-treated planktonic cells, in contrast, were dramatically irregular and roughened, which increased in severity with concentration, reflecting increased cell wall damage (cells treated with a representative dAMP, RP554, are shown in Fig. 2).

In biofilms, fluconazole-treated cells showed only a coating with a film-like or granular substance, likely residual extracellular matrix. The surfaces of the *Candida* cells within the biofilm, containing some pseudohyphal or hyphal cells, became roughened, corrugated, and flattened upon treatment with RP554, increasing with increasing concentrations, indicating severe cell wall damage and implying cellular perturbation via membrane disruption.

### Limited cytotoxicity to mammalian cells.

**FIG 3:**
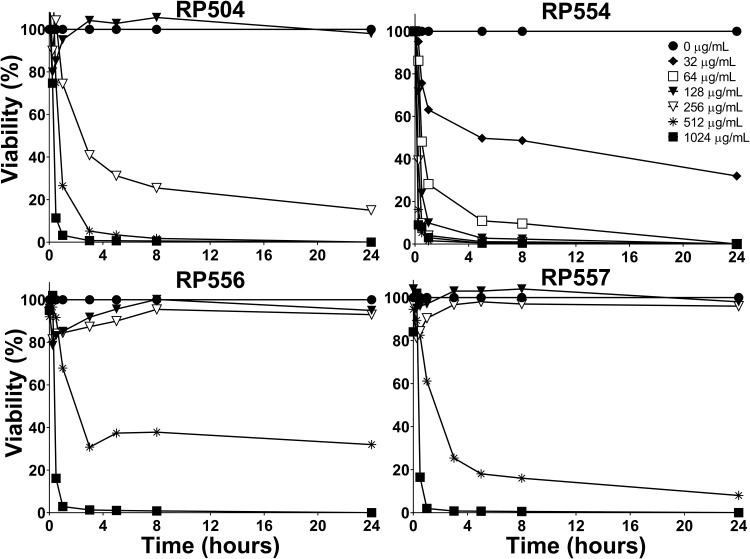
RP556 and RP557 exhibit minimal mammalian cell toxicity. Cellular toxicity was noninvasively assayed using bioluminescent human keratinocytes and viability was assayed using an IVIS Lumina imaging system. Cells were plated at 1 × 10^4^ cells/well, adhered overnight, dAMPs added, and bioluminescence evaluated over 8 h. Concentrations were performed in triplicate, and data is from two independent experiments.

AMP development has been hampered by unwanted toxicity to mammalian cells. The dAMPs evaluated here exhibited varying degrees of cytotoxicity, with RP556 and RP557 exhibiting limited toxicity as demonstrated by using both a noninvasive continuous bioluminescence assay (Fig. 3) and a lactate dehydrogenase (LDH) membrane disruption release assay. The results from the bioluminescence live imaging assays and the LDH assay were similar (not shown). The 10% lethal dose (LD_10_), which is the concentration required to kill 10% of the human keratinocytes, determined by bioluminescence live imaging, following 8 h incubation, for RP504, RP554, RP556, and RP557 was 143, 19, 276, and 287 μg/ml, respectively, correlating to 58, 10, 128, and 134 μM.

### Treatment results *in vivo*.

**FIG 4:**
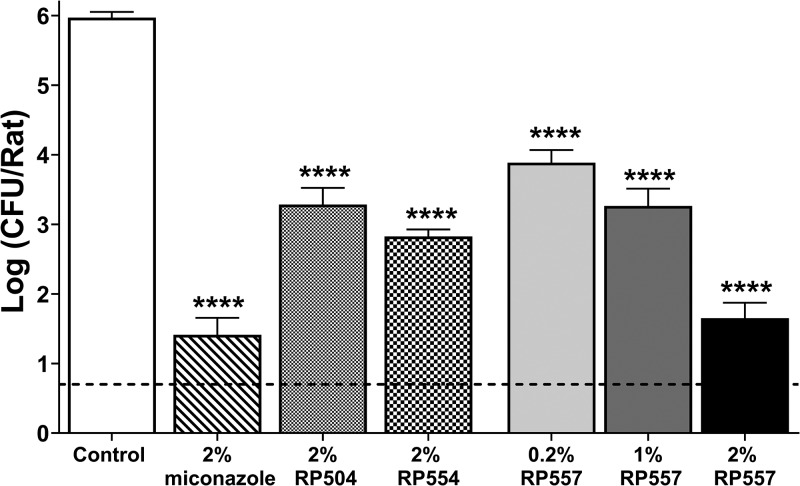
dAMP topical treatment reduces vulvovaginal candidiasis in a rodent model. Effects of miconazole (2%, *n* = 10), RP504 (2%, *n* = 5), RP554 (2%, *n* = 5), and RP557 (0.2%, *n* = 5; 1%, *n* = 5; 2%, *n* = 10) were evaluated and compared to those of the controls (*n* = 10). The C. albicans (ATCC 44858) vaginal infection model involved oophorohysterectomized Wistar rats with CFU evaluated at day 5. On day 0, the animals were inoculated IVG with C. albicans at 1.5 × 10^7^ CFU/rat (0.1 ml/rat). dAMPs and miconazole were administered IVG at 0.1 ml/rat BID at 8-h intervals starting from 48 h after infection for a total of three sequential days. CFU were evaluated on day 5 with the limit of detection (LOD, dashed lines) being 0.7 CFU/rat. Data represent mean ± standard error (SE) with significant difference defined as *P* < 0.0001 (****) compared to that of the control as determined by one-way ANOVA followed by Dunnett’s test.

The activity of intravaginally (IVG) administered dAMPs was evaluated in an immunosuppressed rat vulvovaginal candidiasis model. Treatment was administered IVG twice daily (BID) starting from 48 h after C. albicans infection for a total of three sequential days. dAMP treatment did not cause any adverse safety concerns as measured by clinical observations, body weight, and histopathology. Histologically, there was no damage to the squamous epithelium lining of the treated vagina. The day 5 CFU results are depicted in Fig. 4. The topical application of 2% RP504, RP554, or RP557 resulted in significant reductions in fungal counts relative to the control group in a rodent VVC model (Fig. 4 [all *P* < 0.0001]), with RP557 being the most potent. The positive control, 2% miconazole, caused a near 4-log reduction in fungal counts, compared to the concurrent control group (*P* < 0.0001). There was no difference in activity between 2% RP557 and 2% miconazole. All evaluated doses of RP557, 0.2, 1, or 2%, caused marked reductions in vulvovaginal candidiasis, with 2% causing the greatest reduction.

## DISCUSSION

The increase in RVVC infections has generated an urgent need for new antifungal agents with novel mechanisms of action that are active against antimycotic-resistant *Candida* and recalcitrant biofilm with a limited likelihood of developing resistance. Designed antimicrobial peptides (dAMPs) are engineered analogs of naturally occurring AMPs that are ubiquitous in nature and provide the first line of defense against invading pathogens.

The dAMPs (RP504, RP544, RP556, and RP557) exhibited broad-spectrum antifungal activity against 46 clinical isolates comprising fluconazole-sensitive and resistant strains of C. albicans, C. glabrata, C. tropicalis, and C. parapsilosis and intrinsically resistant C. krusei. The dAMPs are commonly fungicidal, as MIC values were similar to MFCs, in contrast to fluconazole which is fungistatic (8). To confirm the mechanism of action via direct membrane perturbation, electron microscopy performed on both planktonic and biofilm C. albicans was consistent with dAMP-mediated fungal effects via membrane perturbation, whereas fluconazole appeared only fungistatic.

AMPs are part of the innate immune response (23). Although there is extensive evidence for AMP actions at microbial membranes, and they bind to membrane sterols (22), there is also evidence that AMPs can bind to target ribosomal subunits; can inhibit synthesis of DNA, RNA, proteins, and macromolecules; and can inhibit respiration, intracellular protein folding, and iron regulation (24–26). These intracellular actions may be relevant to antifungal action, as AMP penetration into fungal cells by many different mechanisms has been shown (27). Particularly relevant to the antifungal actions that we demonstrate in our studies, AMPs have also been shown to bind cell wall glucan and thus possibly interfere with cell wall synthesis (28). At the host level, AMPs can act as signaling molecules and immune regulators (23). The myriad of actions described may be alternative antifungal actions to a membrane-active dAMP property or augment it.

Biofilm has been debated to be a critical component of RVVC pathogenicity and recalcitrance (4, 14, 28–30, 43). Biofilms are intrinsically resistant to currently approved antifungal treatment, and compared to planktonic cells, usually require drug concentrations many-fold the corresponding MIC values for biofilm eradication (31). In contrast, the dAMPs exhibited the ability to affect biofilm at concentrations far less than those required for planktonic isolates, particularly RP554 and RP557 with preformed biofilm activity quotients of 0.125 and 0.5, respectively. A similar phenomenon has been observed with the naturally occurring AMP LL-37 on Pseudomonas aeruginosa biofilms. LL-37 was found to affect biofilm formation by inhibiting bacterial cell attachment, stimulating twitching motility, and affecting two quorum sensing systems, thereby downregulating genes necessary for biofilm growth (32). A possible limitation of the biofilm studies is that yeast cells could potentially have left the biofilm or been removed from the biofilm during the procedures and that these planktonic yeast cells may have had a different susceptibility than the remaining adherent cells.

An efficacious antifungal must be able to affect fungi and not host cells, thereby decreasing off-target safety concerns. The development of a clinically viable AMP has previously been hampered by unwanted toxicity to mammalian host cells at therapeutic doses (33); therefore, the cytotoxicity of dAMPs was evaluated using human keratinocytes transfected with a bioluminescent reporter gene to enable real-time assessment of mammalian cell viability. RP556 and RP557 exhibited limited toxicity with LD_10_ concentrations of 276 and 287 μg/ml (128 and 134 μM) following 8 h incubation, followed by RP554 and RP504 at 143 and 19 μg/ml (58 and 10 μM), respectively. The amounts of dAMP required to kill 10% of keratinocytes was orders of magnitude higher than that needed to inhibit *Candida* in both planktonic and biofilm forms.

The topical application of dAMPs was effective in reducing C. albicans in a rodent VVC model. The efficacy of intravaginally administered RP504, RP554, and RP557 was compared to miconazole and vehicle control in an immunosuppressed rat VVC rodent model. A recent study indicated oral fluconazole to be ineffective in this model (34). RP557 was more effective than RP554 and RP504; however, there was no difference in activity between RP557 and miconazole. It could be clinically more beneficial to prescribe RP557 over miconazole for RVVC, as resistance rates to miconazole have increased from 2.4% in 2006 to 59.8% in 2013 for C. albicans (35). Among non-*albicans* species, miconazole was resistant against 25.6% and 73.1% of C. glabrata and C. krusei species, respectively. As RP557 yielded the best *Candida* cell specificity over mammalian cells, was potent in both preformed and formed biofilm, and was active in the first VVC study, it was chosen to undergo an *in vivo* dose-response evaluation. An increasing dose-dependent reduction in fungal burden occurred, with 2% RP557 yielding a statistically significant reduction (*P* < 0.0001) compared to that of the respective vehicle control. Miconazole (2%) and 2% RP557 generated similar activity, with the results of both antimicrobials consistent with the first study.

Based on the initial encouraging antimicrobial results observed in planktonic isolates and biofilm cultures for *Candida* and *in vivo* antimicrobial activity following topical application in a rodent vaginal candidiasis model, we believe that dAMPs are potential therapeutic agents for the treatment of RVVC infections and have less susceptibility for developing microbial resistance. Further studies will also be undertaken to investigate potential immunomodulatory, resident bacterial microflora and both long-term safety and therapeutic effects of RP557 treatment. Preclinical development is under way to evaluate RP557 as a potential therapeutic for the treatment of vulvovaginal candidiasis.

## MATERIALS AND METHODS

### Peptides.

Four dAMPs, whose amino acid sequences are depicted in Table 1, were synthesized by AmbioPharm (North Augusta, SC) using a synthetic, solid-phase peptide synthesis scheme. Peptide purity was >96% as assayed by high-performance liquid chromatography/mass spectroscopy. The dAMPs evaluated here were rationally designed from tachyplesin, a disulfide β-sheet antimicrobial peptide found in hemocytes of the horseshoe crab (Tachypleus tridentatus) (36). Iterative structure activity modifications to the peptide sequences included amidation of the C terminus, use of non-natural amino acids with replacement of lysine by ornithine, and optimization of charge density and hydrophobicity.

### Source of *Candida* isolates.

*Candida* clinical isolates were obtained from the California Institute for Medical Research (CIMR), San Jose, CA. The isolates selected for dAMP evaluation included C. albicans (5/10), C. glabrata (5/4), C. tropicalis (5/2), C. parapsilosis (5/1), and C. krusei (0/3), with the fractions given representing susceptible (S) and resistant (R) (S/R), responses to fluconazole, as defined by CLSI criteria (37). Other *Candida* clinical isolates were tested against fluconazole for comparative purposes. C. albicans (ATCC 44858) for the rat vaginal candidiasis assay was obtained from the American Type Culture Collection (Rockville, MD, USA).

### MIC and MFC determination.

Inhibition of planktonic growth (MIC) was assessed for the 46 clinical isolates by the broth macrodilution method in RPMI 1640 using the Clinical and Laboratory Standards Institute (CLSI) methodology (37), with breakpoints used for fluconazole as per CLSI. The range of concentrations tested included 0.5 to 32 μg/ml comprising 2-fold serial dilutions.

The minimum fungicidal concentration (MFC) was evaluated for all 4 dAMPs on a fluconazole-susceptible and a fluconazole-resistant strain of C. albicans, C. glabrata, C. tropicalis, and C. parapsilosis, and a resistant C. krusei, for a total of 9 isolates. These isolates were randomly selected within each of those groups. Evaluations were performed by subculturing aliquots immediately following determination of the MIC and defined as killing of ≥96% of inoculum (38).

### Induction of resistance.

The long-term effects of RP554, RP556, and RP557 were evaluated on planktonic growth of C. tropicalis 17-25 by stepwise exposure to increased dAMP concentrations. Inocula of 5 × 10^2^ cells/ml were incubated in the presence of each dAMP for a minimum of 48 h or for the time necessary for the fungus to reach half the density of an untreated concurrent control or a maximum of 7 days (whichever was longer). The rationale behind the necessity to increase the time between passages is in order to collect a sufficient inoculum for the next passage as the population decreases in successive passages owing to drug action. At dAMP concentrations where the fungus was so inhibited that it did not reach the density described after 7 days of cultivation, the described inoculum was passed from the drug-containing tube to the next higher dAMP concentration, as well as to a control tube not containing the drug. Once the fungus reached, in a peptide concentration, the density described, an inoculum was prepared and passed to a 2-fold higher drug concentration as well as to a control tube not containing dAMP. This exercise was performed over a 2-fold range of concentrations from 0.06 to 32 μg/ml.

### Biofilm inhibition.

Candida albicans 17-88, 5 × 10^5^ cell/ml, was allowed to form biofilm for 16 h, washed three times with 200 μl phosphate-buffered saline (PBS), and subsequently challenged immediately or following 24 h with the test articles to assess effects on biofilm formation or preformed biofilm inhibition, respectively (39). The dAMPs or fluconazole were diluted in RPMI 1640 with a final well concentration of 64, 32, 16, or 8 μg/ml and compared to control wells containing only RPMI. Each condition contained 3 replicates. Following a total incubation time of 40 h, the wells were washed three times with 200 μl PBS, and then 200 μl XTT (2,3-bis-(2-methoxy-4-nitro-5-sulfophenyl)-2H-tetrazolium-5-carboxanilide salt) and menadione solution were added to each well. The plates were incubated at 37°C for 2 h and absorbance read, and data presented, at 490 nm for assessment of inhibition of metabolism. The endpoint was a statistically significant reduction compared to an untreated concurrent control.

### Scanning electron microscopy.

Planktonic C. albicans isolates were morphologically evaluated following treatment at a drug concentration equal to their MIC level. Established (preformed) C. albicans biofilm was evaluated following a 24-h incubation with 8, 16, 32, or 64 μg/ml of agents. Samples were processed for scanning electron microscopy as previously described (39, 40). In brief, C. albicans biofilm samples were cultured on 8-well chamber slides and treated with fluconazole (32 μg/ml) and RP554 (16, 32, and 64 μg/ml) as described above before being fixed and processed for SEM (40). Planktonic samples were treated with fluconazole (1 μg/ml) and RP554 (16 μg/ml) to approximate their respective drug inhibitory concentrations before being lightly pelleted and fixed similarly in 4% paraformaldehyde with 2% glutaraldehyde in 0.1 M sodium cacodylate buffer. Aliquots (100 μl) of yeast suspension were allowed to settle onto poly-l-lysine-coated coverslips for 12 min before osmium tetroxide postfixation, dehydration, and hexamethyldisilazane treatment as described for biofilm samples. After mounting onto aluminum stubs and gold coating using an Edwards S150A sputter coater to enhance conductivity, samples were visualized with a Zeiss Merlin field emission scanning electron microscope (Carl Zeiss Microscopy, Germany) using 3 kV accelerating voltage, 90 to 100 pA probe current, and both InLens secondary electron and secondary electron 2 detection. Images were captured in TIFF files using a pixel averaging noise reduction algorithm and 2,048 by 1,536 pixel store resolution. Evaluations were performed in duplicate.

### Bioluminescent human keratinocytes mammalian cytotoxicity.

Noninvasive and real-time monitoring of dAMP mammalian cell toxicity was performed using bioluminescent human keratinocytes. A luciferase stably expressed human keratinocyte (HaCaT; AddexBio Technologies, Inc., San Diego, CA) cell line was created by transfection with a luciferase gene (RediFect Red-Fluc-Puromycin, catalog number CL596002; Perkin Elmer, Waltham, MA, USA) (41). The resultant multiplicity of infection was 40:1.

To evaluate mammalian cell viability, the bioluminescent keratinocytes were added, at 10^4^ cells per well (100 μl volume), to black-walled, 96-well plates. Growth medium was supplemented with 150 μg/ml d-luciferin. Plates were incubated overnight at 37°C. dAMPs, formulated in water, were 2-fold serially diluted from a starting concentration of 2,048 μg/ml in growth medium supplemented with 150 μg/ml d-luciferin. The dilution series was performed in triplicate in a separate 96-well plate. The peptide dilutions, 100 μl, were transferred to the keratinocyte plate, resulting in a 200 μl total volume. Each dAMP was studied in triplicate. Results presented are from two independent experiments. Imaging was performed at 0, 15, and 30 min and then 1, 3, 5, and 8 h after the addition of the dAMP and compared to concurrently run dAMP-free control wells, using an IVIS Lumina imaging system (Caliper Life Sciences, Inc., Hopkinton, MA, USA). An exposure time of 1 min, open filter, f-stop 1 was utilized, and data analysis was performed using the Living Image software program (version 4.3; Caliper Life Sciences, Inc.). To confirm the cytotoxicity obtained using the bioluminescence assay correlated with a conventional metabolic release assay, a lactate dehydrogenase (LDH) cell viability assay (LDH-Glo cytotoxicity assay; Promega) was also utilized. The dAMP concentration required to kill 10% of cells was determined using GraphPad Prism 7 (GraphPad Software, San Diego, CA, USA).

### Animals.

All animals received care in compliance with the “Guide for the Care and Use of Laboratory Animals: Eighth Edition” (National Academies Press, Washington, DC, 2011) in an AAALAC-accredited ABSL-2 laboratory vivarium.

### Rat vulvovaginal candidiasis model.

Oophorohysterectomized and immunosuppressed female Wistar rats were vaginally infected with Candida albicans strain ATCC 44858. Estrogen levels in the rats were first regulated by subcutaneous (s.c.) administration of estradiol at 10 mg/kg for 3 days before infection (day 3) and at 4 mg/kg s.c. on day 4. The animals were also immunosuppressed with the addition of dexamethasone, 2 mg/liter, to the drinking water. Dexamethasone treatment started 3 days before infection and continued through study termination. On day 0, the animals were inoculated intravaginally (IVG) with C. albicans at 1.25 × 10^7^ CFU/rat (0.1 ml/rat). dAMPs, RP554 and RP557, formulated in 2% hydroxypropyl methylcellulose 4000 (HPMC) (Sigma-Aldrich, MA, USA), a semisynthetic, inert, viscoelastic polymer used extensively as an ophthalmic lubricant, and reference standard mycoderin (2% miconazole; Gyno-mycoderin cream, Bowlin, Taiwan) were administered IVG at 0.1 ml/rat twice daily (BID) at 8-h intervals starting from 48 h after infection (day 2) for a total of three sequential days. Body weights were taken predose and at termination.

Animals were euthanized on day 5. Vaginal lavage was performed twice with 0.2 ml PBS, and the lavage fluid was pooled and C. albicans counts, CFU/rat, determined by plating 10-fold dilutions of samples to Sabouraud agar plates followed by incubation and enumeration. After vaginal lavage fluid collection, the vaginal tissues were collected from vehicle and the treatment groups, fixed in 10% formalin, stained with hematoxylin and eosin, and then evaluated (5 sections of vagina in duplicate) by a board-certified veterinary pathologist.

### Statistical analysis.

Quantitative data were expressed as mean ± standard error or mean ± standard deviation. Statistical analysis was performed using GraphPad Prism 7 (GraphPad Software, San Diego, CA, USA). Unpaired *t* tests were used to compare differences between two groups. For multiple comparisons, a one-way analysis of variance (ANOVA) followed by Dunnett’s *post hoc* analysis was used. Statistical significance is considered at a *P* value of <0.05.
